# Annual acknowledgement of manuscript reviewers

**DOI:** 10.1186/s12960-016-0105-z

**Published:** 2016-03-09

**Authors:** Mario Roberto Dal Poz

**Affiliations:** University of the State of Rio de Janeiro, Rio de Janeiro, Brazil

## Abstract

The editors of *Human Resources for Health* would like to thank all our reviewers who have contributed to the journal in Volume 13 (2015).

**Matilda Aberese-Ako**

Ghana

**Mauro Henrique Abreu**

Brazil

**Elizabeth Adams**

Ireland

**Orvill Adams**

Canada

**Adam Ahmat**

Congo

**Ducksun Ahn**

Korea, Republic Of

**Mohamad Alameddine**

Lebanon

**Hasanat Alamgir**

USA

**Jackeline Alger**

Honduras

**Robert Alhassan**

Ghana

**Fernanda Almeida**

Brazil

**Alvaro Alonso-Garbayo**

UK

**Alla Alsharif**

Australia

**Alaa Alsharif**

Australia

**Ghanim Y M Alsheikh**

UK

**Woldekidan Kifle Amde**

South Africa

**Claire Anderson**

UK

**Truong Anh Thu**

Viet Nam

**Laura Antonietti**

Argentina

**Edson Araujo**

USA

**Ricardo Arcencio**

Brazil

**Angel Asúnsolo**

Spain

**Elsheikh Badr**

Sudan

**Jane Ball**

UK

**Allan Barbosa**

Brazil

**Emma Barnes**

UK

**Maria Soledad Barria**

Chile

**Robert Basaza**

Uganda

**Ernesto Báscolo**

Argentina

**Ken Bassett**

Canada

**Kisalaya Basu**

Canada

**N Batura**

UK

**Lorne Bellan**

Canada

**Sara Bennett**

USA

**David Benton**

Switzerland

**Miteshkumar Bhanderi**

India

**Kavita Bhatia**

India

**Aarushi Bhatnagar**

India

**Posy Bidwell**

UK

**Said Bodur**

Turkey

**Marc Bonenberger**

Switzerland

**Noemi Bordoni**

Argentina

**Ivy Bourgeault**

Canada

**Susan Bradley**

UK

**Ruairi Brugha**

Ireland

**James Buchan**

Australia

**Yvonne Buys**

Canada

**Johann Cailhol**

South Africa

**Jennifer Callaghan-Koru**

USA

**Brian Cameron**

Canada

**Maureen Canavan**

USA

**Dora Cardaci**

Mexico

**Thomas Chacko**

India

**Yasmin Chandani**

Kenya

**Yotamu Chirwa**

Zimbabwe

**Cari Clark**

USA Minor Outlying Islands

**Helen Cleak**

Australia

**Rhonda Cockerill**

Canada

**Sandra Cole**

USA

**Giorgio Cometto**

Switzerland

**John Connell**

Australia

**Sandro Contini**

Italy

**Jonathan Cowpe**

UK

**Elaine Daily**

USA

**Anita Alero Davies**

Switzerland

**Kenneth De Camargo**

Brazil

**Willemijn De Graaf-Ruizendaal**

Netherlands

**Francis Deng**

USA

**Shrey Desai**

India

**Ibadat Dhillon**

USA

**Roland Dimaya**

USA

**Yan Ding**

Germany

**Beata Dobrowolska**

Poland

**Carmen Dolea**

Switzerland

**Delanyo Dovlo**

Namibia

**Norbert Dreesch**

Austria

**Carl-Ardy Dubois**

Canada

**Christine Duffield**

Australia

**Gilles Dussault**

Portugal

**Tom Dyer**

UK

**Michelle Dynes**

USA

**Shah Ebrahim**

UK

**Matt Edwards**

UK

**Leonie Ellis**

Australia

**Isabel Emmerick**

USA

**Boniface Ikenna Eze**

Nigeria

**Jane Ferguson**

UK

**Paulo Ferrinho**

Portugal

**Linda Fogarty**

USA

**Luiz Eduardo Fonseca**

Brazil

**Luciana De Barros Correia Fontes**

Brazil

**Alfredo Fort**

USA

**Sandra Fortes**

Brazil

**Allison Foster**

USA

**Allison Annette Foster**

USA

**Silvia Franzetti**

Argentina

**Seble Frehywot**

USA

**Ines Fronteira**

Portugal

**Diana Frymus**

USA

**Suzanne Fustukian**

UK

**Marie-Pierre Gagnon**

Canada

**Caroline Gaither**

USA

**Jenny Gallagher**

UK

**Ana Gama**

Portugal

**Juan García-Ubaque**

Colombia

**Gulin Gedik**

Switzerland

**Laurent Gerbaud**

France

**Beatriz Gl Valcarcel**

Spain

**Irene Glinos**

Belgium

**Helen Goodyear**

UK

**Susan Gordon**

Australia

**William Greene**

USA

**Gustavo Gusso**

Brazil

**Bui Ha**

Viet Nam

**Rohini J Haar**

USA

**Amy Hagopian**

USA

**Mythri Halappa**

India

**Paula Hancock**

UK

**Christine Hancock**

USA

**Mark Hann**

UK

**Piya Hanvoravongchai**

Thailand

**Janet Harris**

UK

**Ivonete Heidemann**

Brazil

**Julie Henderson**

Australia

**Alison Hernández**

Sweden

**Peter Heywood**

Australia

**Peter Hill**

Australia

**David Hipgrave**

Australia

**Silvia Hirsch**

Argentina

**Miriam Hirschfeld**

Israel

**Stephen Hodgins**

USA

**Steven Justin Hoffman**

Canada

**Vincent Homburg**

Netherlands

**Chee Peng Hor**

Malaysia

**Kun Huang**

China

**Sara Hughes**

Singapore

**Niamh Humphries**

Ireland

**Anna-Karin Hurtig**

Sweden

**Kazuo Inoue**

Japan

**Jehu Iputo**

South Africa

**Masato Izutsu**

Japan

**Verughese Jacob**

USA

**Christel Jansen**

Netherlands

**Edgar Jarillo**

Mexico

**Wanda Jaskiewicz**

USA

**Katrine Jensen**

Denmark

**Yaping Jin**

Canada

**Benjamin Johns**

USA

**Jennifer Johnson**

USA

**Patrick Kadama**

Uganda

**Amer Kaissi**

USA

**László Kalabay**

Hungary

**Simon Kamau**

Kenya

**Francis Williams Kamwendo**

Malawi

**Sumit Kane**

Netherlands

**Srinivasan Kannan**

India

**Rebecca Katz**

USA

**Sheila Keane**

Australia

**Sosena Kebede**

USA

**Sunil Khanna**

USA

**Yohannes Kinfu**

Australia

**Maxwell Kligerman**

USA

**Maryse Kok**

Netherlands

**Adam Koon**

UK

**Lisbeth Kristiansen**

Sweden

**Marieke Kroezen**

Belgium

**Judith Kulig**

Canada

**Saravana Kumar**

Australia

**Ramesh Kumar**

Pakistan

**Rafael Kurtzbart**

Argentina

**Talma Kushnir**

Israel

**Gaetan Lafortune**

France

**Mylene Lagarde**

UK

**Michel Landry**

USA

**Luís Lapão**

Portugal

**Sarah Larkins**

Australia

**Elysia Larson**

USA

**Carole-Lynne Le Navenec**

Canada

**Yeong Yeh Lee**

Malaysia

**David Legge**

Australia

**Uta Lehmann**

South Africa

**Lia Levin**

Israel

**Miguelhete Lisboa**

Mozambique

**Jenny Liu**

USA

**Alba Llop-Gironés**

Spain

**Elsbet Lodenstein**

Netherlands

**Sérgio Lopes**

Portugal

**Vera Lucia Luiza**

Brazil

**John Luque**

USA

**Cristiani Machado**

Brazil

**Adrian Mackenzie**

Canada

**Timothy Mackey**

USA

**Diana Madden**

Australia

**Elizabeth Madigan**

USA

**Carinne Magnago**

Brazil

**Ozayr Mahomed**

South Africa

**Claudia Maier**

USA

**Dilip Singh Mairembam**

India

**Ana Maria Malik**

USA

**Mats Malqvist**

Sweden

**Ogenna Manafa**

Ireland

**Kate Mandeville**

UK

**Ghulam Farooq Mansoor**

Afghanistan

**Ludmila Marcinowicz**

Poland

**Kanchan Marcus**

Australia

**Susana Margulies**

Argentina

**Tim Martineau**

UK

**Grant Martsolf**

USA

**Keith Masnick**

Australia

**Leah Masselink**

USA

**Andrea Veronica Mastrangelo**

Argentina

**Maria Mathews**

Canada

**Verona Mathews**

South Africa

**Josue Mbonigaba**

South Africa

**Willy Mccourt**

UK

**Matthew Mcgrail**

Australia

**Pamela Mcquide**

Namibia

**Joanne Mcveigh**

Ireland

**Araya Abrha Medhanyie**

Ethiopia

**Hugo Mercer**

Argentina

**Edward Mills**

Canada

**Mario Monteiro**

Brazil

**Anthony Montgomery**

Greece

**Jean Moore**

USA

**Joanna Morrison**

UK

**Frederick Mugisha**

Uganda

**Amin Muhammad Gadit**

Canada

**Wilbroad Mutale**

Zambia

**Janet Myers**

USA

**Lucio Naccarella**

Australia

**Takashi Nagata**

Japan

**Papreen Nahar**

UK

**Joel Negin**

Australia

**Anne Nesbitt**

Sierra Leone

**Jonathan Newbury**

Australia

**Ha Nguyen**

USA

**Gustavo Nigenda**

Mexico

**Mimi Niles**

USA

**Mwansa Annette Nkowane**

Switzerland

**Godswill Nnaji**

Nigeria

**Charles Normand**

Ireland

**Mary Patricia Nowalk**

USA

**Humphreys Nsona**

Malawi

**Jennifer Nyoni**

Congo

**Jerry Okal**

Kenya

**Larry Olsen**

USA

**Pauline Oosterhoff**

Netherlands

**Carole Orchard**

Canada

**Francisco Ortega**

Brazil

**Belinda O'sullivan**

Australia

**Susanne Ozegowski**

Germany

**Nermin Ozgulbas**

Turkey

**Ligia Paina**

USA

**Elisabeth Paul**

Belgium

**Mark Pauly**

USA

**Matthew Pearce**

USA

**Ann Pederson**

Canada

**Marina Peduzzi**

Brazil

**Senga Pemba**

Tanzania

**Ha Pham**

Viet Nam

**Pudtan Phanthunane**

Thailand

**Anastas Philalithis**

Greece

**Yogan Pillay**

South Africa

**Victor Piriz**

Uruguay

**Patricia Pittman**

USA

**Evgeniya Plotnikova**

UK

**Kaja Polluste**

Estonia

**María Pozzio**

Argentina

**Ari Probandari**

Indonesia

**Eduardo Benjamin Puertas**

Ecuador

**Mary Purkis**

Canada

**Weerasak Putthasri**

Thailand

**Lal Rawal**

Bangladesh

**Stephen Reid**

South Africa

**Michelle Remme**

UK

**Thomas Ricketts**

USA

**Jemimah Ride**

Australia

**Monika Riedel**

Austria

**Patricia Riley**

USA

**Laetitia Rispel**

South Africa

**Bjarne Robberstad**

Norway

**Priscilla Robinson**

Australia

**Jose L. Rocha**

Spain

**Wesley Rohrer**

Saudi Arabia

**Janetta Roos**

South Africa

**Ellen Roskam**

Switzerland

**Arie Rotem**

Australia

**Giuliano Russo**

Portugal

**Gayle Rutherford**

Canada

**Dana Ryan**

Canada

**Mandy Ryan**

UK

**Bukola Salami**

Canada

**Fabiano Saldanha Gomes De Oliveira**

Brazil

**Domenico Salvatore**

Italy

**Delia Sanchez**

Uruguay

**Teresa Sanchez-Sagrado**

France

**Milena Santric Milicevic**

Serbia

**Eric Sarriot**

USA

**María Cecilia Scaglia**

Argentina

**Marta Schaaf**

USA

**Richard Scheffler**

USA

**Andreas Schmid**

Germany

**Philip Seidenberg**

USA

**Tarun Sen Gupta**

Australia

**Walter Sermeus**

Belgium

**P Ravi Shankar**

Nepal

**Amani Shao**

Tanzania

**John Shaw**

New Zealand

**Zubin Shroff**

Switzerland

**Jonathan Sicsic**

France

**Mohsin Sidat**

Mozambique

**Martin Silberman**

Argentina

**Daudi Simba**

Tanzania

**Donald Simpson**

USA

**Amani Siyam**

Switzerland

**Marion Slack**

USA

**Martin Smith**

South Africa

**Jeffrey Soar**

Australia

**Julie Sochalski**

USA

**Nils Gunnar Songstad**

Norway

**Michael Spencer**

USA

**Allison Squires**

USA

**Barbara Stilwell**

USA

**Sabine Stordeur**

Belgium

**Roger Strasser**

Canada

**Tara Tancred**

UK

**Shenglan Tang**

USA

**Akhenaten Tankwanchi**

USA

**Katherine Taylor**

UK

**Conor Teljeur**

Ireland

**Petra Ten Hoope-Bender**

Switzerland

**Kea Tijdens**

Netherlands

**Vandana Tripathi**

USA

**Eralda Turkeshi**

Albania

**Liina-Kaisa Tynkkynen**

Finland

**Graciela Uriburu**

Argentina

**Peter Van Bogaert**

Belgium

**Hendrik P. Van Dalen**

Netherlands

**Anke Van Der Kwaak**

Netherlands

**Paula Vassallo**

Malta

**Vanesa Vazquez Laba**

Argentina

**Marko Vujicic**

USA

**Qun Wang**

China

**Rachel J Waxman**

USA

**William Weeks**

USA

**Madeleen Wegelin**

Netherlands

**William Willis**

USA

**Kim Wilson**

USA

**Nancy Winslade**

Canada

**Christiane Wiskow**

Switzerland

**Matthias Wismar**

Belgium

**Kelley Withy**

USA

**Timothy Wood**

Australia

**Scott Wright**

USA

**Ansgar Wuebker**

Germany

**Kaspar Wyss**

Switzerland

**Mezgebu Yitayal**

Ethiopia

**Beibei Yuan**

Sweden

**Junhua Zhang**

China

**Rose Zulliger**

USA

**Joseph Zulu**

Zambia

**Prisca Ac Zwanikken**

Netherlands

